# Increased numbers of CD5^+^CD19^+^CD1d^high^IL-10^+^ Bregs, CD4^+^Foxp3^+^ Tregs, CD4^+^CXCR5^+^Foxp3^+^ follicular regulatory T (TFR) cells in CHB or CHC patients

**DOI:** 10.1186/s12967-014-0251-9

**Published:** 2014-09-09

**Authors:** Li Wang, Jinpeng Qiu, Lei Yu, Xiaoli Hu, Pingwei Zhao, Yanfang Jiang

**Affiliations:** Key Laboratory of Zoonosis Research, Ministry of Education, the First Hospital of Jilin University, Changchun, 130021 China; Department of infectious Disease, the fourth hospital of Harbin medical University, Harbin, 150001 China; Department of Infection, Heilongjiang Province Hospital, Harbin, 150056 China; Department of Pediatrics, the First Affiliated Hospital of Jiamusi University, Jiamusi, 154002 China

**Keywords:** Chronic hepatitis C (CHC), Chronic hepatitis B (CHB), Breg, Follicular regulatory T (TFR), T follicular helper (TFH), Forkhead box protein 3 (Foxp3), IL-10, HBsAg

## Abstract

**Background:**

IL-10^+^ regulatory B (Bregs), CD4^+^Foxp3^+^ regulatory T (Tregs), and CD4^+^CXCR5^+^Foxp3^+^ follicular regulatory T (TFR) cells regulate the progression of infection disease. This study aimed at examining how those cells associated with the development of chronic hepatitis B (CHB) and chronic hepatitis C (CHC) in a Chinese population.

**Methods:**

The numbers of circulating IL-10^+^ Bregs, Tregs and TFR cells in 31 CHC, 58 CHB patients and 22 healthy controls (HC) were examined by flow cytometry. The potential association of those cells with clinical measures was analyzed.

**Results:**

The numbers of CD5^+^CD19^+^CD1d^high^IL-10^+^ Bregs, Tregs and TFR cells and the levels of serum IL-10, IFN-γ and IL-2 in the CHB, and IL-10 and IFN-γ in the CHC patients were significantly higher than that in the HC (p < 0.05). Furthermore, the numbers of circulating IL-10^+^ Bregs and the levels of serum IL-10, but not other cytokines tested were positively correlated with the levels of serum HBV DNA and ALT in the HBeAg^−^ CHB patients as well as HCV RNA and ALT in CHC patients. Additionally, the numbers of circulating TFR cells were positively correlated with the levels of serum HBV DNA and ALT in the CHB patients as well as HCV RNA and ALT in the CHC patients.

**Conclusions:**

Increased numbers of circulating IL-10^+^ Bregs and TFR cells are associated with poor virus eradication and liver injury in CHB and CHC patients. Furthermore, the levels of serum IL-10 is associated with the hepatic flares.

## Introduction

Hepatitis B virus (HBV) or hepatitis C virus (HCV) infection remains a serious health problem in the world, particularly in China. Currently, HBV or HCV infection affects about 350 or 170 million people worldwide and 93 or 30 million people in China [1]. Persistent infection with HBV or HCV can cause chronic hepatitis B (CHB) or chronic hepatitis C (CHC), respectively, and many patients with CHB or CHC eventually progressively develop liver cirrhosis, hepatocellular carcinoma (HCC) and end-stage liver disease [2,3]. More importantly, the pathogenic process of CHB and CHC is still not fully understood. Previous studies have shown that poor T cell immunity is associated with the pathogenesis of CHB and CHC [4,5]. However, the regulation of T cell immunity against HBV or HCV during the process of CHB or CHC has not been fully understood.

It is well known that forkhead box protein 3 (Foxp3)^+^ Tregs can inhibit immune responses [6-9]. Emerging studies have shown that Tregs can inhibit virus-specific T cell immunity in the pathogenesis of CHB or CHC [6-8,10,11]. In addition, CD19^+^CD5^+^CD1d^high^IL-10^+^ Bregs and CD1d^+^CD5^+^ B10 cells can also inhibit T cell immunity [12-16] and regulate autoimmunity, infection and cancer [17-22]. A recent study indicates that higher levels of serum IL-10 and a higher frequency of circulating Bregs in CHB patients are associated temporally with hepatic flares in Europeans [23]. However, little is known about whether and how the numbers of circulating IL-10^+^ Bregs are associated with clinical pathogenic features in Chinese patients with CHB or CHC.

CXCR5^+^CD4^+^ T follicular helper (TFH) cells are important for the formation of germinal center and humoral responses [24]. Interestingly, recent studies have shown that a subset of Foxp3 + Bcl6+ TFH cells (defined as follicular regulatory T (TFR) cells) share many characteristics with Tregs and inhibit immune responses [25-27]. Currently, there is little information about the numbers of TFR cells in humans and there is no report about the numbers of circulating TFR cells in patients with CHB or CHC and what the potential role TFR cells play in the pathogenesis of CHB or CHC. In addition, the potential relationship among Tregs, IL-10^+^ Bregs, and TFR cells has not been explored in Chinese patients with CHB or CHC.

In the present study, we characterized the numbers of TFR cells, IL-10^+^ Bregs and Tregs in 31 patients with CHC, 58 patients with CHB and 22 gender-, age-, and ethnicity-matched healthy controls (HC). We found that the numbers of TFR cells, Tregs, CD5^+^CD19^+^CD1d^high^IL-10^+^ Bregs and the levels of serum IL-10 in patients with CHB or CHC were significantly greater than those in the HC. Furthermore, the numbers of CD5^+^CD19^+^CD1d^high^IL-10^+^ Bregs and the levels of serum IL-10 were correlated positively with the levels of serum HBV DNA or HCV RNA in the HBeAg^−^ CHB and CHC patients, respectively. In addition, the numbers of Tregs, Bregs and TFR cells in CHB and CHC patients were also correlated with positively the levels of serum HBV DNA, HCV RNA and ALT in the CHB or CHC patients, respectively. These data suggest that these regulatory cells inhibited antigen-specific immunity, contributing to the pathogenesis of CHB or CHC.

## Methods

### Study subjects

A total of 31 patients with CHC and 58 patients with CHB were recruited sequentially at the inpatient service of the First Hospital of Jilin University (Changchun, China) from September 2009 to June 2013. The experimental protocol was established, according to the ethical guidelines of the Helsinki Declaration and was approved by the Human Ethics Committee of Jilin University, China. Written informed consent was obtained from individual participants. Individual subjects with CHB were diagnosed, based on the evidence of serum positive HBsAg and HBV DNA (HBV-DNA) for at least 6 months. Subjects were excluded for the CHB group if she/he had hepatitis C, D or G, HIV infection, autoimmune hepatitis or metabolic liver disease, HCC, receiving immunosuppressive therapy, or antiviral therapy within 6 months. Patients with CHC were diagnosed, according to positive detection of serum anti-HCV antibodies and HCV-RNA (>100 IU/ml) for at least 6 months. Individuals were excluded from the CHC group if she/he had serum positive for HBsAg, hepatitis D or G virus infection and anti-HIV, decompensated cirrhosis, HCC, a history of alcohol abuse, seizure disorders, or active autoimmune disease. Another 22 gender-, age-, and ethnicity-matched healthy subjects with normal values of liver function tests and negative serological results of viral and autoimmune liver diseases served as the HC. All subjects denied being a drug user or having been exposed to hepatotoxins, based on their knowledge [28]. The demographic and clinical characteristics of the patients are summarized in Tables 1 and 2.

**Table 1:** **The demographic and clinical characteristics of participants**

**Parameters**	**HC (n = 22)**	**CHB (n = 58)**	**CHC (n = 31)**
Age (years)	42 (22–74)	48 (25–76)	54 (34–74)
Gender: Female/male	6/16	17/41	9/22
ALT (U/L)	27 (13–38)	125 (13–2463)*	57 (13–201)*
AST (U/L)	25 (10–36)	106 (21–1932)*	67 (16–173)*
HCV RNA level (Log_10_ IU/ml)	NA	NA	6.42 (3.69–8.60)*
HBV DNA level (Log_10_ IU/ml)	NA	7.26 (5.03–9.98)*	NA
WBC (×10^9^/L)	5.57 (4.3–9.7)	7.26 (4.60–12.5)*	7.13 (4.8–12.3)*
Lymphocytes (×10^9^/L)	1.70 (1.02–2.7)	2.14 (1.36–3.12)	1.92 (1.45–2.48)
HBsAg (IU/ml)	NA	29.63 (2.97–306.68)*	NA
HBsAb (mIU/ml)	NA	1.12 (0–391.73)*	NA
HBeAg (S/CO)	NA	2.38 (0.03–540.84)*	NA
HBeAb (S/CO)	NA	1.36 (0.02–24.57)*	NA
HBcAb (S/CO)	NA	1.04 (0.03–19.17)*	NA
HCVAb (S/CO)	NA	NA	7.28 (1.09–34.6)*
HBeAg (+/−)	NA	36/22	NA
Disease duration (month)	NA	100.8 (14.4–324)	12 (7–36)

Data shown are real case number or median (range). Normal values: ALT: <40 IU/L; AST: <40 IU/L, WBC: (4.0-10.0) × 10^9/L, Lin#: (0.8-4.0) × 10^9/L, HBsAg: <0.050 IU/mL, HBsAb: <10.000 mIU/mL, HBeAg: <1.0000 S/CO, HBeAb: <1.000 S/CO, HBcAb: <1.000 S/CO, HCVAb: <1.000 S/CO. HC: healthy control; CHB: Chronic hepatitis B, CHC: Chronic hepatitis C; HBsAg: hepatitis B surface antigen; HBsAb: hepatitis B surface antibody; HBeAg: hepatitis B e antigen; HBeAb: hepatitis B e antibody; HBcAb: hepatitis B core antibody. *P <0. 05 vs. the HC.

**Table 2:** **The demographic and clinical characteristics of HBeAg** ^**-**^ **and HBeAg** ^**+**^ **CHB patients**

**Parameters**	**HBeAg** ^**−**^	**HBeAg** ^**+**^
Age (years)	46 (25–76)	48 (29–68)
Gender: Female/male	9/13	8/28
ALT (U/L)	145.5 (21–2463)	86 (13–1803)
AST (U/L)	90 (26–1932)	121.5 (21–1838)
HBV DNA level (Log_10_ IU/ml)	6.52 (5.25–8.23)	7.62 (5.03–9.98)*

*P <0. 05: the HBeAg^+^ CHB vs. the HBeAg^-^ CHB.

### Clinical index assay

The clinical data of individual subjects enrolled in the study were collected from the hospital records. These data included age, sex, and laboratory tests. Their blood samples were subjected to routine laboratory tests, including the levels of serum alanine transaminase (ALT) and spartate aminotransferase (AST) by Beckman-coulter LX-20 Biochemistry Automatic Analyzer (Beckman-coulter, California, USA). The levels of serum HBV-DNA (the detection limitation ≥50 IU/ml) and HCV-RNA (the detection limitation ≥100 IU/ml) were tested by fluorescence-based quantitative PCR and RT-PCR using specific kits, according to the manufacturers’ instruction (Haoyuan Diagnostics, Shanghai, China). The concentrations of serum anti-HCV, HBsAg, HBsAb, HBeAg, HBeAb and HBcAb were detected by a dynamic laser light scattering turbidimetry on a protein analyer (Abbott Laboratories, North Chicago, USA).

### Flow cytometry analysis

Venous blood samples were collected from individual subjects and peripheral blood mononuclear cells (PBMCs) were isolated by density-gradient centrifugation using Ficoll-Paque Plus (Amersham Biosciences, Little Chalfont, UK). PBMCs at 5 × 10^5^/tube were stained in duplicate with APC-anti-CD4 (R&D Systems), FITC-anti-CD25 or isotype-matched controls (BD BioSciences, San Jose, USA) for 30 min. Furthermore, PBMCs (5 × 10^5^/tube) were also stained in duplicate with Alexa Fluor 647-anti-CXCR5, PE-Cy7-anti-CD4 (BD PharMingen, San Diego, USA) for 30 min. After being washed with Flow Cytomestry Staining Buffer Solution (e-Bioscience), the cells were fixed and permeabilized by Cytofix/Cytoperm solution (e-Bioscience). Subsequently, PBMCs were stained intracellularly with PE-anti-FoxP3 (e-Bioscience) for the detection of CD4^+^Foxp3^+^ and CD4^+^CXCR5^+^Foxp3^+^ T cells, respectively. At least 50,000 events were analyzed by FlowJo software (v7.6.2).

To characterize IL-10^+^ Bregs, PBMCs were cultured in duplicate in 24-well plates (10^6^ cells/well) and stimulated with 50 ng/mL of phorbol myristate acetate (PMA), 1.0 μg/mL of ionomycin, and 50 ng/mL lipopolysaccharide (LPS, Sigma-Aldrich, St Louis, MO, USA) in 10% fetal bovine serum (FBS) RPMI-1640 medium (complete medium) for 2 h at 37°C in 5% CO_2_ and then exposed to Brefeldin A (GolgiPlug; BD Biosciences, San Jose, CA, USA) for an additional 4 h. The cells were harvested, washed with ice-cold PBS, and stained with APC-anti-CD19, PerCP-anti-CD5, and PE-anti-CD1d (eBiosciences). The immunostained cells were fixed with 4% paraformaldehyde for 30 min at room temperature and permeabilized with 0.5% saponin in 10% FBS in PBS for 30 min at room temperature. After being washed, the cells were stained intracellularly with FITC-anti-IL-10 (eBiosciences) and analyzed by flow cytometry. At least 50,000 events per sample were analyzed.

### Quantification of IL-10 by enzyme-linked immunosorbent assay (ELISA)

The concentrations of serum IL-10 in individual subjects were measured using an ELISA kit, according to manufacturer’s instructions (Sunny ELISA Kit, Mutisciences, Hangzhou, China). Briefly, individual sera at 1:2 dilutions were subjected in triplicate to ELISA analysis, and the concentrations of serum IL-10 in individual samples were calculated, according to the standard curve established using recombinant human IL-10 provided. The limitation of detection for serum IL-10 was 0.59 pg/ml.

### Cytometric bead array (CBA) analysis of serum cytokines

The concentrations of serum cytokines (IL-17A, IFN-γ, TNF-α, IL-6, IL-4, and IL-2) were determined by CBA using the CBA kit, according to the manufacture’s protocol (CBATM, BD Biosciences, San Joes, USA). Individual samples were quantified in duplicate on a FACSCalibur cytometry (BD Biosciences), and the data were acquired using the CellQuestPro and analyzed by the CBA software (Becton Dickinson) [29].

### Statistical analysis

Data are expressed as median and range or individual mean values. The difference between the groups was analyzed by Fisher exact test or Mann–Whitney U nonparametric test using the SPSS 19.0 software. The relationship between variables was evaluated using the Spearman rank correlation test. A two-side P value of < 0.05 was considered statistically significant.

## Results

### Increased numbers of CD5 + CD19 + CD1dhighIL-10+ Bregs and elevated levels of serum IL-10 in CHB or CHC patients

To investigate the potential role of IL-10^+^ Bregs, TFR cells and Tregs in CHB or CHC patients, 31 patients with CHC, 58 patients with CHB and 22 gender-, age-, and ethnicity-matched healthy subjects were recruited. There was no significant difference in the distribution of age and gender between the patients and HC (Tables 1 and 2). As expected, all patients had disease duration more than 6 months, consistent with their chronic state. In comparison with that in the HC, abnormally higher levels of serum ALT, AST, positive HBV DNA, HCV RNA, anti-HCV, HBsAg, HBeAg, HBsAb, HBeAb, and HBcAb were detected in CHB or CHC patients, respectively. In addition, significantly greater numbers of WBC, but similar numbers of lymphocytes were detected patients with CHB or CHC in this population. More importantly, the levels of serum HBV DNA were significantly higher in the HBeAg^+^ CHB patients than that in the HBeAg^−^ CHB patients.

**Figure 1:**
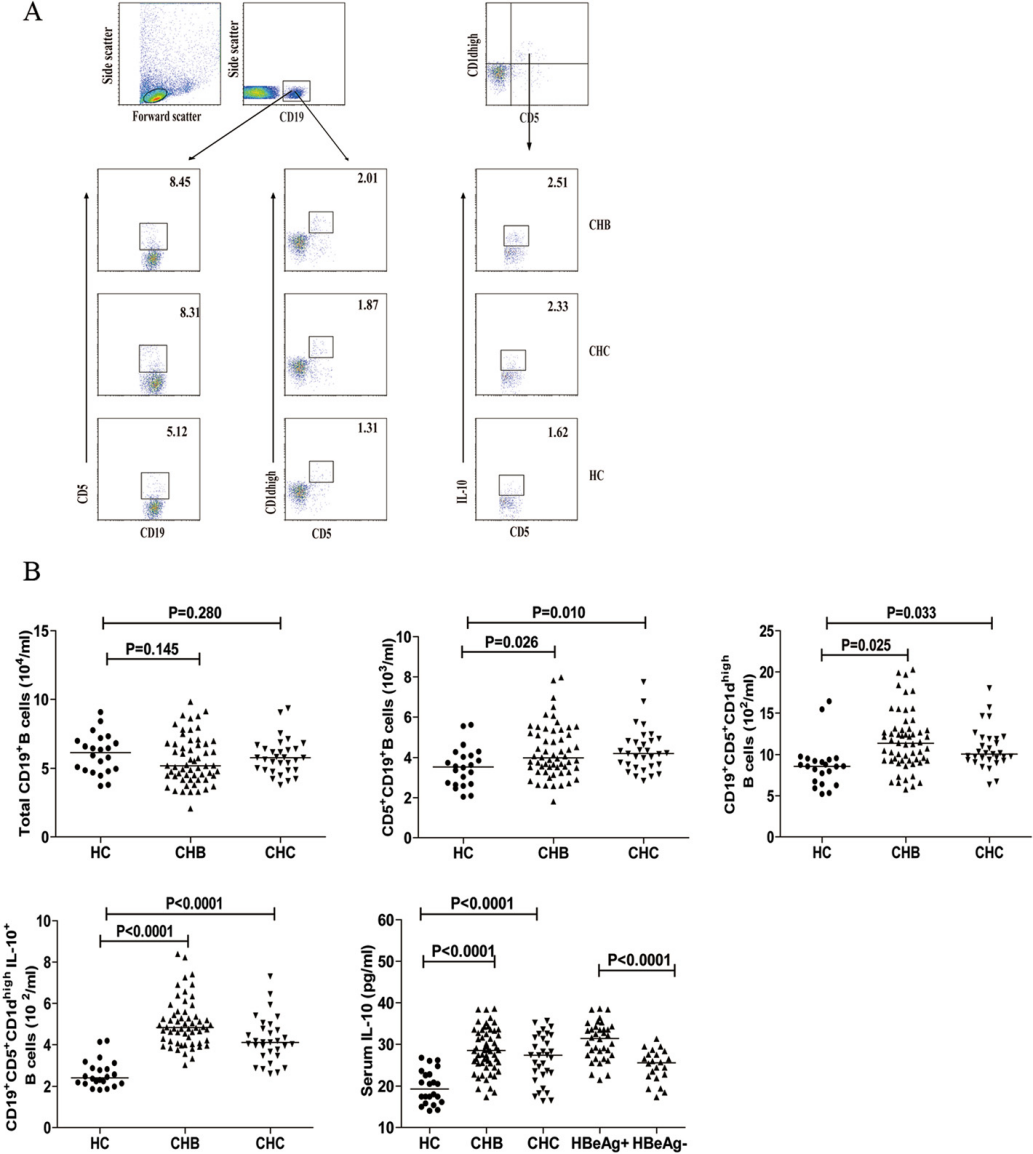
**Flow cytometry analysis of the numbers of circulating IL-10** ^**+**^ **B cells.** PBMCs from individual subjects were plated in 24-well plates (10^6^ cells/well) and stimulated in duplicate with 50 ng/mL of PMA, 1.0 μg/mL of ionomycin, and 50 ng/mL of LPS in complete RPMI-1640 medium for 2 h at 37°C in 5% CO_2_ followed exposing to Brefeldin A for another 4 h. The cells were then harvested, washed with ice-cold PBS, and stained with APC-anti-CD19, PerCP-anti-CD5, and PE-anti-CD1d. The immunostained cells were fixed and permeabilized with 0.5% saponin. After being washed, the cells were stained intracellularly with FITC-anti-IL-10 and analyzed by flow cytometry. The cells were first gated on living lymphocytes and then on CD19^+^ B cells for further analysis of total CD19^+^ B cells, CD5^+^CD19^+^, CD5^+^CD19^+^CD1d^high^ B cells, CD5^+^CD19^+^CD1^dhigh^IL-10^+^ Bregs. The isotype-matched antibodies served as controls. The levels of serum IL-10 in individual subjects were analyzed by ELISA. Data are representative FACS charts and expressed as the mean values of individual subjects and the difference between groups was analyzed by the Kruskal–Wallis test. **(A)** Flow cytometry analysis; **(B)** quantitative analysis of the numbers of total CD19^+^ B cells, CD5^+^CD19^+^, CD5^+^CD19^+^CD1d^high^ B cells, CD5^+^CD19^+^CD1d^high^IL-10^+^ Bregs and the levels of serum IL-10. The horizontal lines indicate the median values for different groups.

We first characterized the numbers of CD5^+^CD19^+^, CD5^+^CD19^+^CD1d^high^ B cells and CD5^+^CD19^+^CD1d^high^IL-10^+^ Bregs in these subjects by flow cytometry analysis. As shown in Figure 1, there was no significant difference in the numbers of circulating CD19^+^ B cells among these three groups of subjects. However, the numbers of CD5^+^CD19^+^, CD5^+^CD19^+^CD1d^high^ B cells and CD5^+^CD19^+^CD1d^high^IL-10^+^ Bregs in both the CHB and CHC patients were significantly greater than those in the HC (p < 0.05) although there was no significant difference in the numbers of CD5^+^CD19^+^CD1d^high^IL-10^+^ Bregs between the CHB and CHC patients. Analysis of serum IL-10 indicated that the levels of serum IL-10 in the CHB and CHC patients were similar and were significantly higher than that in the HC (p < 0.0001). Stratification analysis revealed that the levels of serum IL-10 in 36 HBeAg^+^ CHB patients were significantly higher than that in 22 HBeAg^−^ CHB patients, consistent with a previous observation [22]. Hence, increased numbers of IL-10^+^ Bregs and elevated levels of serum IL-10 existed in patients with CHB or CHC.

**Figure 2:**
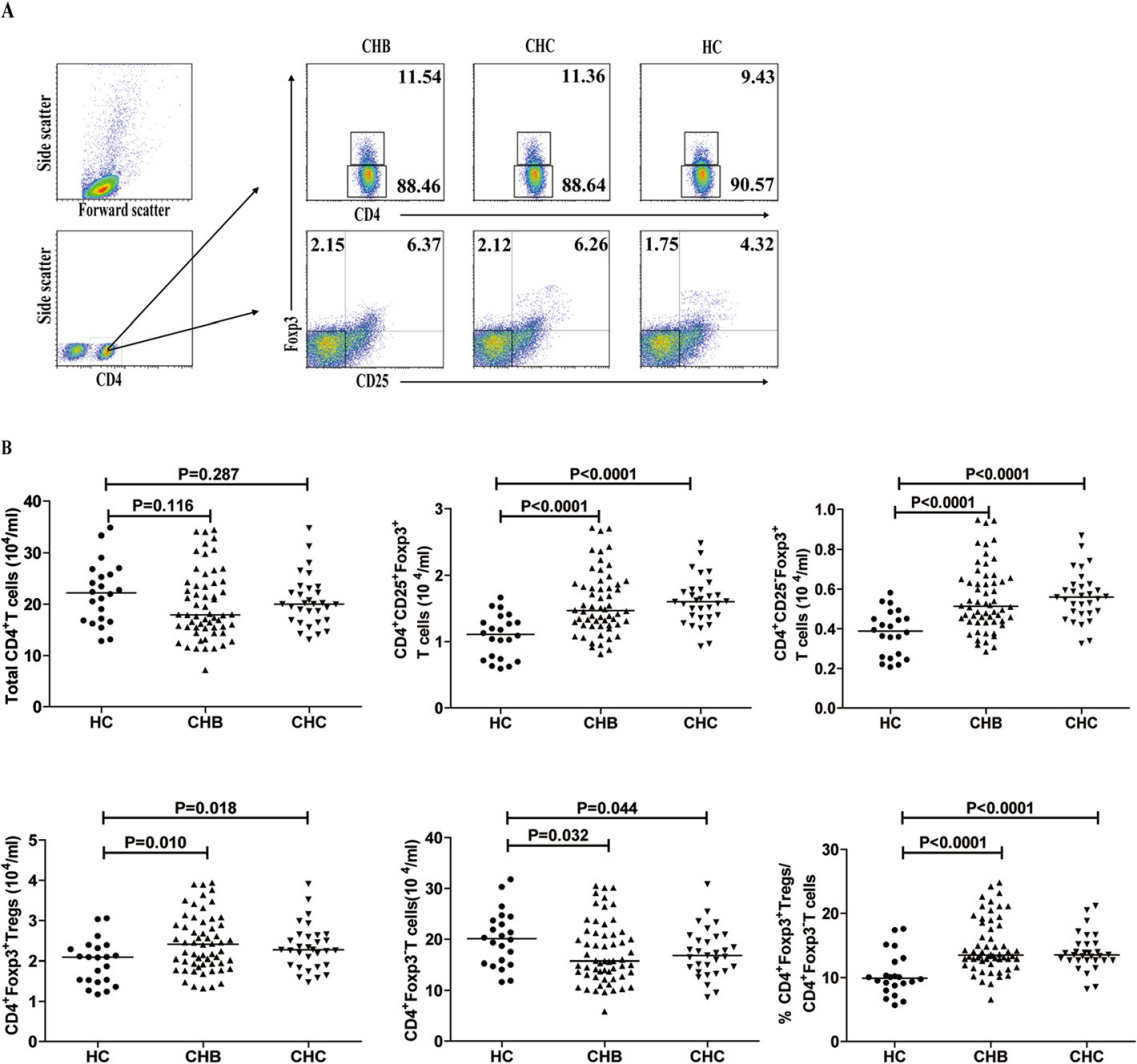
**Flow cytometry analysis of the numbers of circulating Tregs.** PBMC at 5 × 10^5^/tube were stained in duplicate with APC-anti-CD4, FITC-anti-CD25 or isotype-matched controls for 30 min. After being washed, the cells were fixed and permeabilized with Cytofix/Cytoperm. The cells were stained with PE-anti-Foxp3 and the numbers of circulating Tregs were characterized by flow cytometry analysis. Data are representative FACS charts and expressed as the mean values of individual subjects and the difference between groups was analyzed by the Kruskal–Wallis test. **(A)** Flow cytometry analysis; **(B)** The numbers of total CD4^+^ T cells, CD4^+^CD25^−^Foxp3^+^, CD4^+^CD25^+^ Foxp3^+^ Tregs, CD4^+^Foxp3^+^ Tregs, CD4^+^Foxp3^−^ effector T cells and the ratios of CD4^+^CD25^+^ Foxp3^+^ Tregs to CD4^+^Foxp3^−^ effector T cells. The horizontal lines indicate the median values for different groups of subjects.

Given that IL-10^+^ Bregs can promote the development of Tregs, we examined the numbers of Tregs in these subjects by flow cytometry. We found that there was no significant difference in the numbers of circulating CD4^+^ T cells among these groups of subjects and CD4^+^CD25^−^Foxp3^+^ and CD4^+^CD25^+^Foxp3^+^ Tregs between the CHB and CHC patients in this population (Figure 2A and B). However, the numbers of circulating CD4^+^CD25^−^Foxp3^+^ and CD4^+^CD25^+^Foxp3^+^ Tregs in the CHB and CHC patients were significantly greater than those in the HC (p < 0.05 for both). In contrast, the numbers of CD4^+^Foxp3^−^ effector T cells in CHB and CHC patients were significantly less than that in the HC (p < 0.05 for both). As a result, the ratios of Tregs to CD4^+^Foxp3^−^ effector T cells in the CHB and CHC patients were significantly greater than that in the HC (p < 0.0001 for both). Interestingly, the numbers of CD4^+^CD25^+^Foxp3^+^ Tregs were positively correlated with the numbers of CD5^+^CD19^+^CD1d^high^IL-10^+^ Bregs in the CHB (R = 0.7213, p < 0.0001) and CHC (R = 0.7432, p < 0.0001) patients. These data indicate that increased numbers of CD5^+^CD19^+^CD1d^high^IL-10^+^ Bregs are associated with increased numbers of Tregs in CHB or CHC patients.

### Increased numbers of circulating TFH and TFR cells in CHB or CHC patients

**Figure 3:**
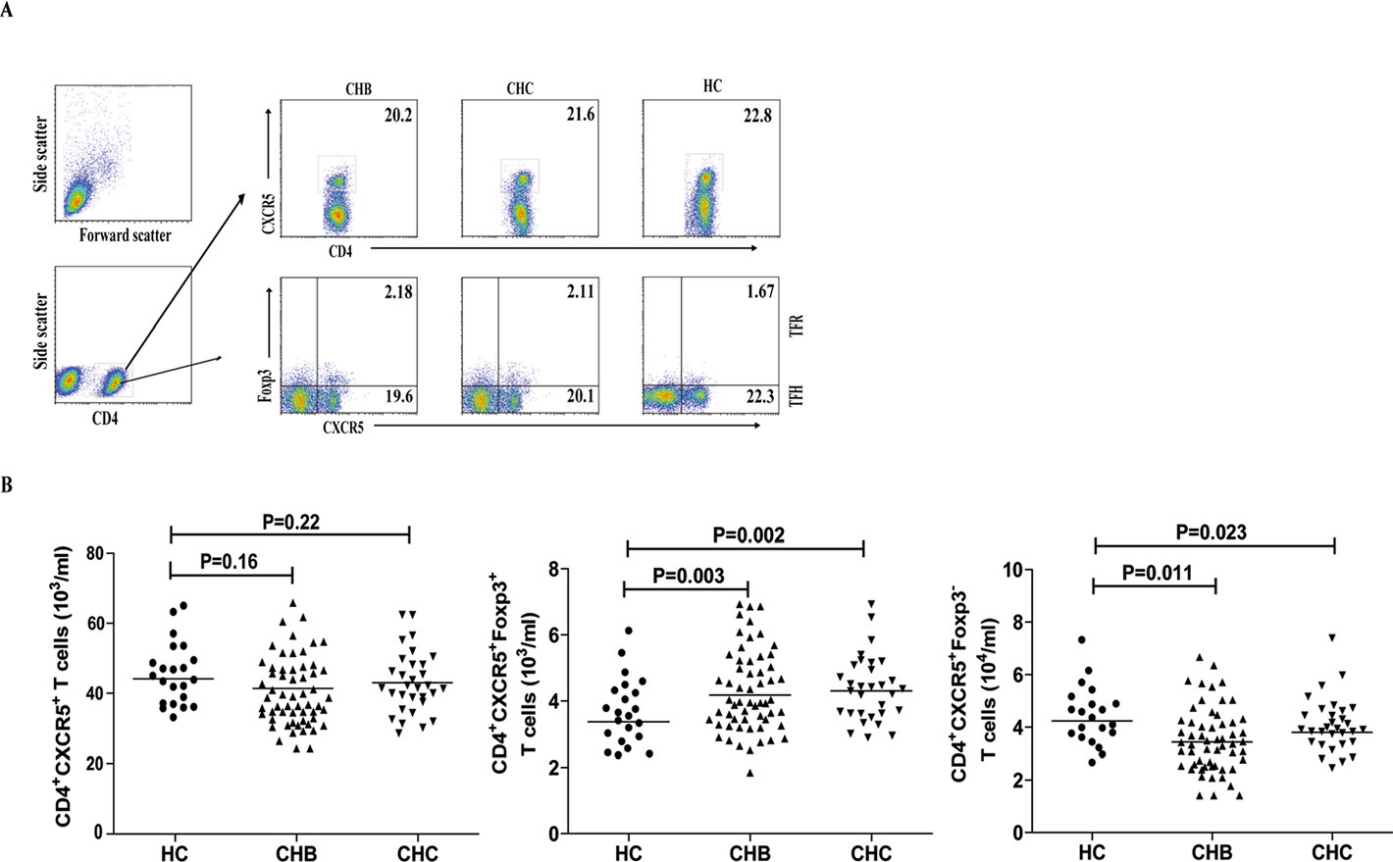
**Flow cytometry analysis of the numbers of circulating TFR and TFH cells.** PBMCs were isolated from individual subjects and were stained in duplicate with Alexa Fluor 647-anti-CXCR5, PE-Cy7-anti-CD4, Alexa Fluor-488-anti-Bcl-6 for 30 min. After being washed, the cells were fixed and permeabilized cells via Cytofix/Cytoperm. The cells were stained with PE-anti-Foxp3 and were characterized by flow cytometry analysis by gating initially on living lymphocytes, and then on CD4^+^ T cells. The isotype-matched antibodies were used as controls. Data are representative FACS charts and expressed as the mean values of individual subjects and the difference between groups was analyzed by the Kruskal–Wallis test. **(A)** Flow cytometry analysis; **(B)** The numbers of CD4^+^CXCR5^+^ T cells, CD4^+^CXCR5^+^Foxp3^−^ (TFH) cells, and CD4^+^CXCR5^+^Foxp3^+^ (TFR) cells. The horizontal lines indicate the median values for individual groups.

TFH cells regulate the formation of germinal center and humoral responses, and are involved in the pathogenesis of CHB and CHC [30,31]. Recently, it has been found that the TFR cells share many characteristics of CD4^+^Foxp3^+^ Tregs, which can express Foxp3 and down-regulate antigen-specific immune responses [27]. To understand the role of TFR cells in the pathogenesis of CHB and CHC, we characterized the numbers of TFR and TFH cells in these subjects by flow cytometry. We found that there was no significant difference in the numbers of CD4^+^CXCR5^+^ T cells, CD4^+^CXCR5^+^Foxp3^+^ TFR and CD4^+^CXCR5^+^Foxp3^−^ TFH cells between the CHB and CHC patients. In contrast, the numbers of TFR cells in the CHB and CHC patients were significantly greater than that in the HC while the numbers of TFH cells were lower in all patients than that in the HC (p < 0.05 for both) in Figure 3. Further analysis indicated that the ratios of TFR to TFH in HBeAg^−^ CHB patients were significantly lower than that in the HBeAg^+^ CHB patients (p = 0.009) and the ratios of TFR to TFH in the CHC patients, but not in the CHB patients, were also significantly lower than that in the HC (p = 0.015, data not shown). In addition, the numbers of TFR cells were positively correlated with the numbers of CD4^+^CD25^+^Foxp3^+^ Tregs and CD5^+^CD19^+^CD1d^high^IL-10^+^ Bregs in both CHB (R = 0.4613, p = 0.0003; R = 0.4245, p = 0.0009) and CHC (R = 0.5417, p = 0.0008; R = 0.4435, p = 0.0100) patients, respectively. Hence, increased numbers of TFR cells existed in the CHB or CHC patients.

**Figure 4:**
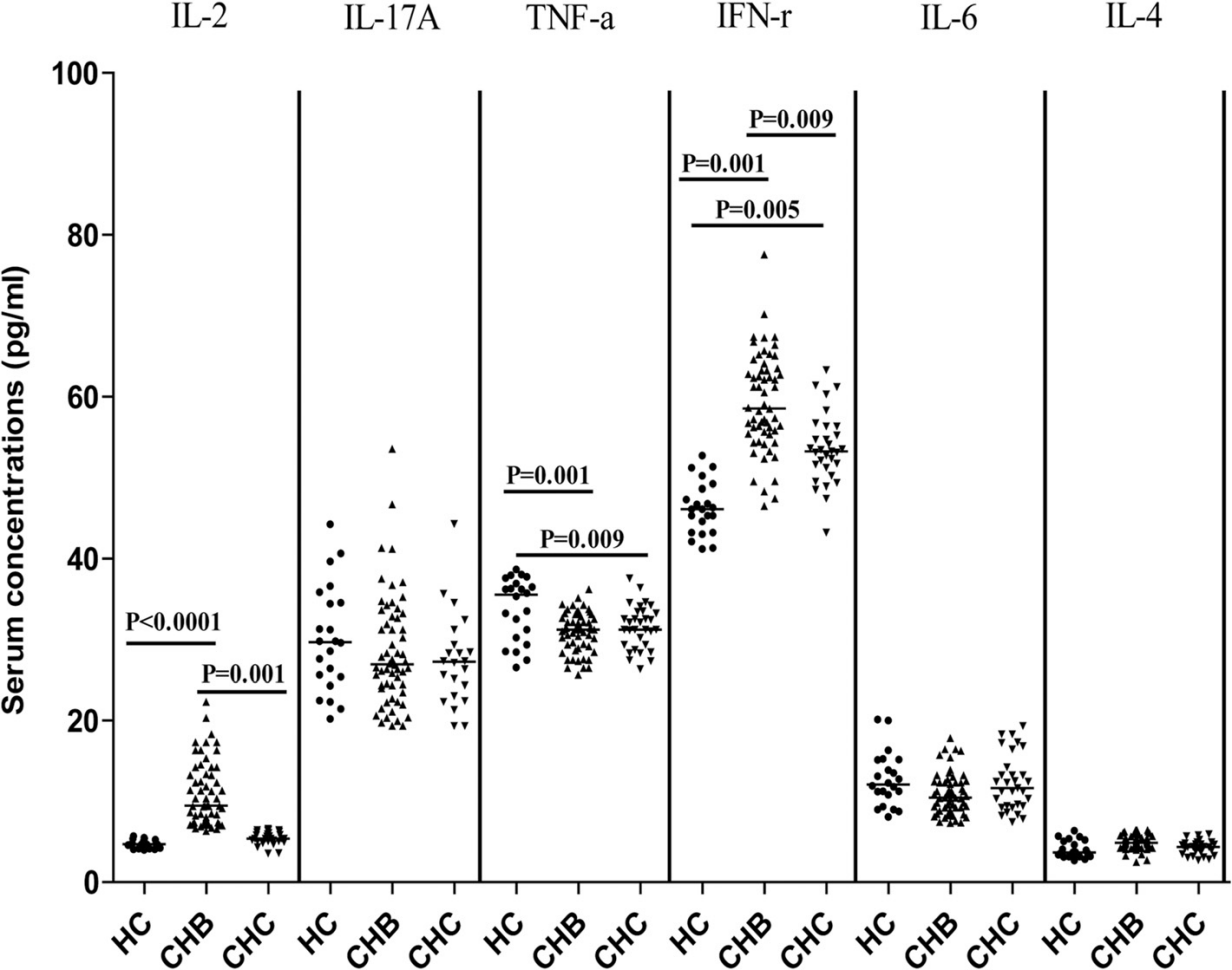
**Analysis of serum cytokines.** The concentrations of serum IL-17A, IFN-γ, TNF-α, IL-6, IL-4 and IL-2 were determined by CBA, and their potential correlations with clinical measures were analyzed. Data are expressed as individual mean values of the different groups of subjects from three separate experiments.

In addition, we further examined the levels of systemic cytokine responses in these subjects by CBA. We found that the concentrations of IL-2 and IFN-γ were significantly elevated in the CHB patients, while only IFN-γ significantly increased in the CHC patients, as compared with that in the HC (Figure 4). Furthermore, the concentrations of serum TNF-α significantly decreased in both CHB and CHC patients, related to that in the HC. However, there was no significant change in the levels of serum IL-4, IL-6, and IL-17A among these groups of subjects in this population, consistent with previous findings [32,33]. Moreover, the levels of serum those cytokines were not significantly correlated with the numbers of Bregs, Tregs, TFR cells or the values of any clinical measures tested in these patients. These data suggest that increased numbers of TFR cells may contribute to the pathogenesis of CHB and CHC.

### Increased numbers of CD5^+^CD19^+^CD1d^high^IL-10^+^ Bregs and elevated levels of serum IL-10 are positively correlated with the values of clinical measures in the HBeAg^−^ CHB and CHC patients

**Figure 5:**
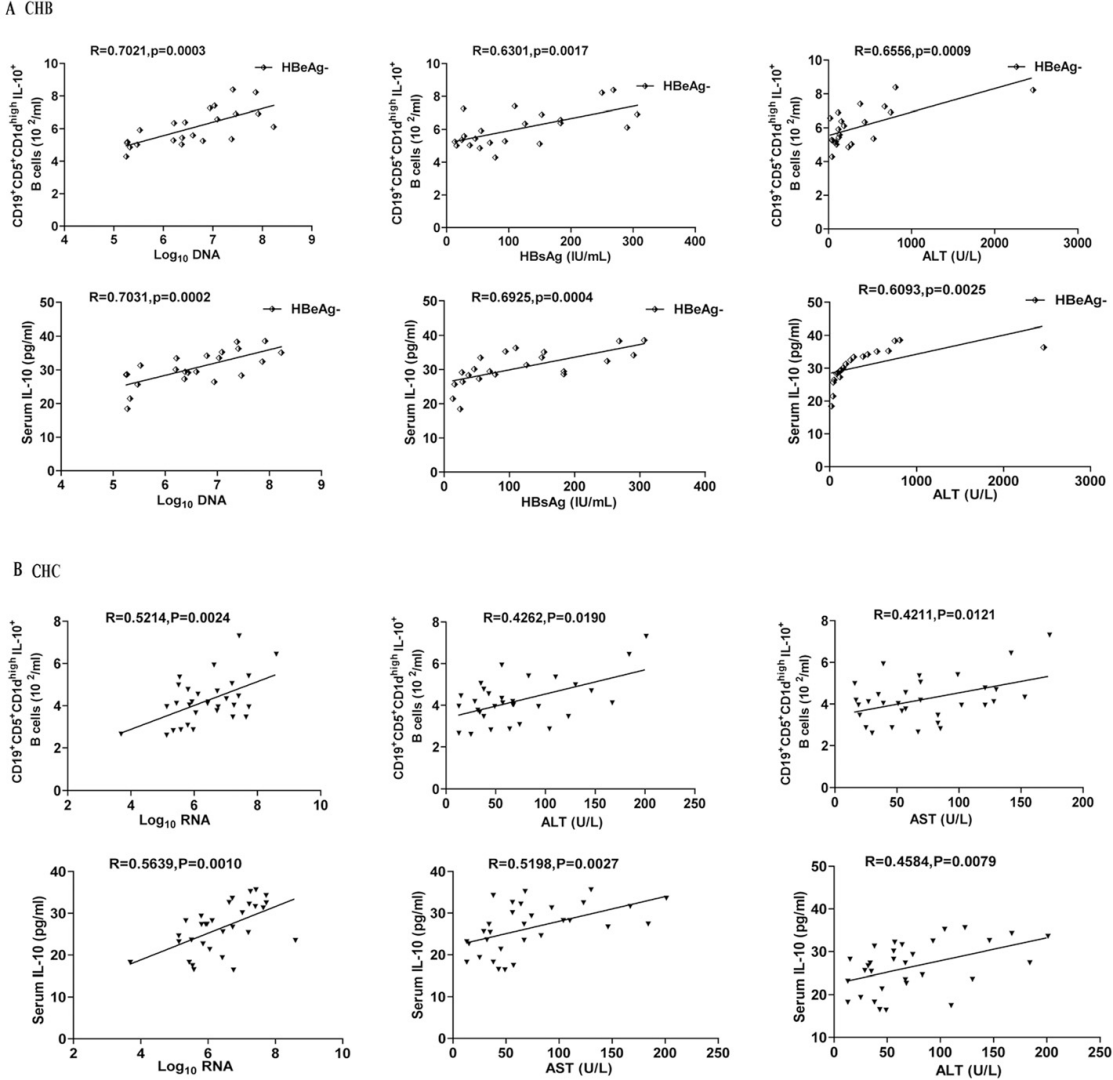
**The correlation analysis between the numbers of CD5** ^**+**^ **CD19** ^**+**^ **CD1d** ^**high**^ **IL-10** ^**+**^ **Bregs and the values of clinical measures in CHB and CHC patients.** The potential correlations between the numbers of CD5^+^CD19^+^CD1d^high^IL-10^+^ Bregs and the values of clinical measures in CHB and CHC patients were analyzed by Spearman’s correlation test. Data shown are the mean values of individual patients. **(A)** The correlation analysis in the HBeAg^−^ CHB patients (n = 22). **(B)** The correlation analyses in the CHC patients (n = 31).

To further understand the significance of increased numbers of IL-10^+^ Breg cells in the pathogenesis of CHB and CHC, we analyzed the potential relationship between the numbers of CD5^+^CD19^+^CD1d^high^IL-10^+^ Bregs and the values of clinical parameters in the CHB or CHC patients. The numbers of circulating CD5^+^CD19^+^CD1d^high^IL-10^+^ Bregs were positively correlated with the concentrations of serum HBV DNA (R = 0.7021, p = 0.0003, Figure 5A), HBsAg (R = 0.6301, p = 0.0017), and ALT (R = 0.6556, p = 0.0009) in the HBeAg^−^ CHB patients. Similarly, the levels of serum IL-10 were correlated positively with the levels of serum HBV DNA (R = 0.7031, p = 0.0002), HBsAg (R = 0.6925, p = 0.0004) and ALT (R = 0.6093, p = 0.0025) in the HBeAg^−^ CHB patients. However, there was no significant association of the numbers of CD5^+^CD19^+^CD1d^high^IL-10^+^ Bregs with the values of other measures tested in CHB patients (data not shown).

Analysis of CHC patients indicated that the numbers of circulating CD5^+^CD19^+^CD1d^high^IL-10^+^ Bregs were correlated positively with the levels of HCV RNA (R = 0.5214, p = 0.0024, Figure 5B), ALT (R = 0.4262, p = 0.0190) and AST (R = 0.4211, p = 0.0121). Similarly, the concentrations of serum IL-10 were correlated positively with the levels of serum HCV RNA (R = 0.5639, p = 0.001), ALT (R = 0.4584, p = 0.0079) and AST (R = 0.5198, p = 0.0027) in the CHC patients. However, there was no significant association of the numbers of CD5^+^CD19^+^CD1d^high^IL-10^+^ Bregs or the levels of serum IL-10 with the values of other measures in CHC patients (data not shown). Apparently, increased numbers of CD5^+^CD19^+^CD1d^high^IL-10^+^ Bregs and elevated serum IL-10 are associated with HBV and HCV replication and liver injury in CHB, especially in the HBeAg^−^ CHB patients, and CHC patients.

### Increased numbers of TFR cells are correlated positively with the levels of serum HBV DNA and HCV RNA in CHB and CHC patients

**Figure 6:**
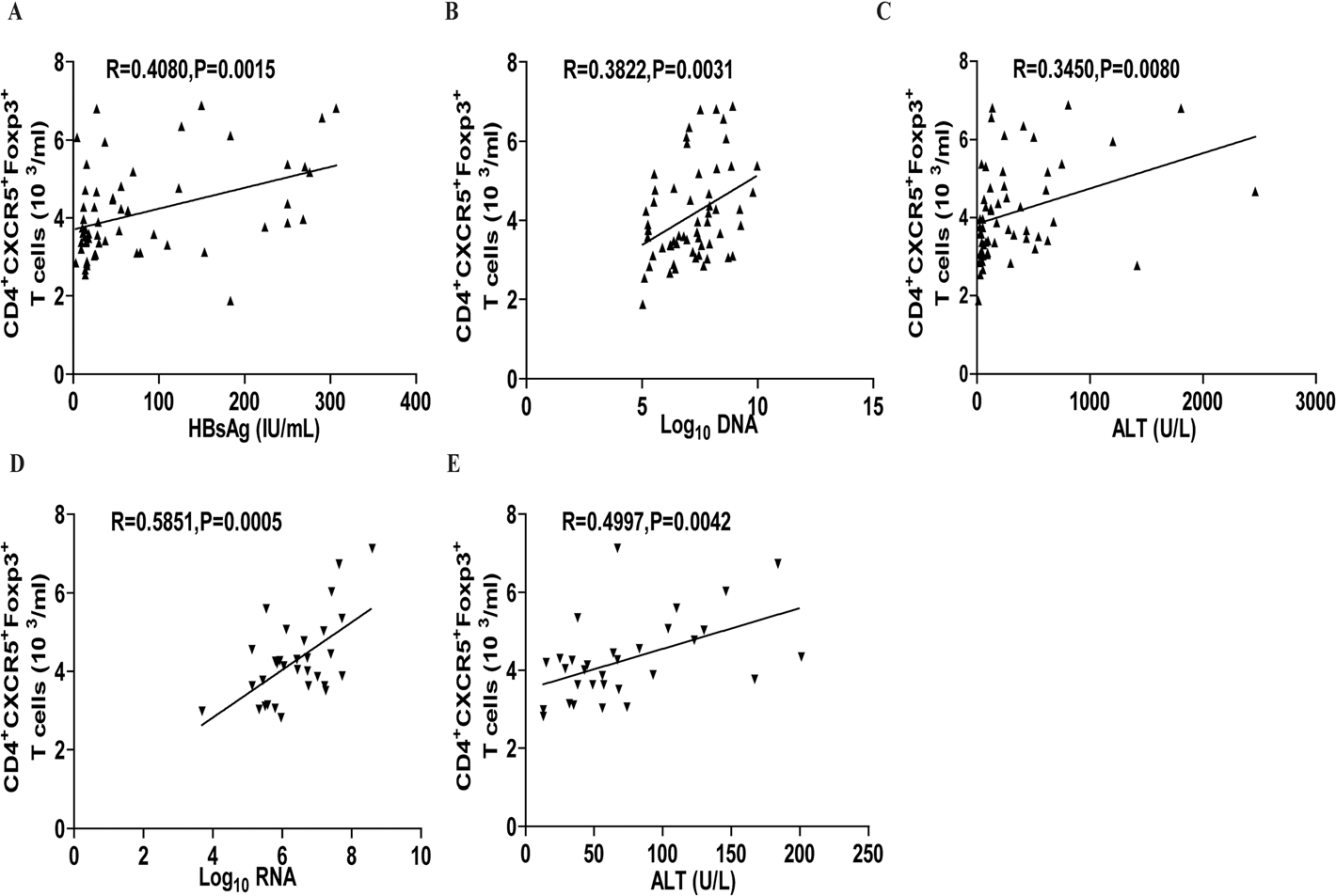
**The correlation analysis between the numbers of TFR cells and the values of clinical measures in CHB and CHC patients.** The potential correlations between the numbers of circulating TFR cells and the values of clinical measures in CHB and CHC patients were analyzed by Spearman’s correlation test. Data shown are the mean values of individual patients. **(A-C)** The numbers of circulating TFR cells are positively correlated with the levels of serum HBV RNA, HBsAg and ALT in the CHB patients (n = 58). **(D-E)** The numbers of circulating TFR cells are positively correlated with the levels of serum HCV RNA and ALT in the CHC patients (n = 31).

To understand the importance of TFR cells, we analyzed the potential association of the numbers of TFR cells with the values of clinical measures in CHB and CHC patients. We found that the numbers of circulating TFR cells were correlated positively with the levels of serum HBsAg (R = 0.4080, p = 0.0015, Figure 6A), HBV DNA (R = 0.3822, p = 0.0031, Figure 6B) and ALT (R = 0.3450, p = 0.008, Figure 6C) in the CHB patients. Similarly, the numbers of circulating TFR cells were correlated positively with the levels of serum HCV RNA (R = 0.5851, p = 0.0005, Figure 6D) and ALT (R = 0.4997, p = 0.0042, Figure 6E) in the CHC patients. However, there was no significant association of the numbers of circulating TFR cells with the values of other measures in the patients of this population (data not shown). Therefore, increased numbers of TFR cells are associated with the virus replication and liver injury in CHB and CHC patients in this population.

## Discussion

Chronic HBV or HCV infection not only causes continual liver damages, but also is associated with the development of cirrhosis and HCC [3,34,35]. Furthermore, recent studies have found that CHB is associated with the development of non-Hodgkin lymphoma (NHL), diffuse large B-cell lymphoma (DLBCL), follicular lymphoma (FL) and T-cell lymphomas (TCL) [36,37]. During the pathogenic process of CHB and CHC, many patients develop immunodeficiency and impaired virus-specific responses. However, the mechanisms underlying virus-specific immune impairment have not been clarified.

Immunoregulatory cells, such as IL-10^+^ Bregs, Tregs and TFR cells have been shown to down-regulate immunity [26,38,39]. Our previous studies and those of others have shown that imbalance of Tregs and effector T cells is associated with the pathogenic process of CHB and poor prognosis of CHB patients. Similarly, increased numbers of circulating Tregs occur in CHC patients, which is associated with weak immune responses against HCV [40,41]. In this study, we examined the numbers of circulating Tregs, IL-10^+^ Bregs and TFR cells in CHB and CHC patients as well as HC. We found that the numbers of circulating IL-10^+^ Bregs and TFR cells, like Tregs, in the CHB and CHC patients significantly greater than those in the HC and they were correlated positively in CHB and CHC patients. Furthermore, the levels of serum IL-10 in the CHB and CHC patients were significantly higher than that in the HC. It is possible that IL-10^+^ Bregs, Tregs and TFR collaborate and help each other, contributing to the development of virus-specific immune impairment and virus poor eradication in CHB and CHC patients.

Bregs play an important role in shaping T cell responses [18,42]. IL-10^+^ Bregs have been shown to inhibit antigen-presenting activity and immune response [43]. In this study, we found significantly increased numbers of circulating IL-10^+^ Bregs and elevated levels of serum IL-10 and that the numbers of circulating IL-10^+^ Bregs were correlated positively with the levels of serum IL-10 in the CHB and CHC patients (data not shown). These data support the notion that IL-10 is an autocrine factor for the development of IL-10^+^ Bregs [38]. Furthermore, it has been shown that IL-10^+^ B cells are associated with the hepatic flares of HBeAg^−^ CHB patients [23]. We found that the numbers of circulating IL-10^+^ Bregs and the levels of serum IL-10 were positively correlated with the concentrations of serum HBV DNA and ALT in the HBeAg^−^ CHB patients and also positively with the concentrations of serum ALT, AST and serum HCV RNA in the CHC patients. Previous studies have shown that T cell immunity, particularly for virus-specific CD8^+^ T cell responses, is crucial for the control of virus replication and liver-injury during the pathogenic process of CHB and CHC [44,45]. Given that IL-10 is a potent inhibitor of T cell immunity, it is possible that IL-10 secreted by Bregs down-regulates T cell immunity and impairs virus eradication. In addition, IL-10^+^ Bregs may promote the development of functional Tregs, impairing anti-virus T cells immunity. Furthermore, we also found that the levels of serum IL-2, IFN-γ and TNF-α were significantly changed in the CHB and CHC patients. However, the precise mechanisms underlying the regulatory effect of IL-10^+^ Bregs and the association of these regulatory cells with the change in the levels of pro-inflammatory cytokines remain to be investigated.

TFR cells are important regulators of TFH and humoral responses [31,46]. We found significantly increased numbers of circulating TFR cells in the CHB and CHC patients and the numbers of circulating TFR cells were correlated positively with the levels of serum HBV DNA, HBsAg and ALT in the CHB patients and with the levels of serum HCV RNA, ALT and AST in the CHC patients. These findings extended our previous observations of significantly higher frequency of TFH cells in CHB and CHC patients [30,31]. Interestingly, we found that the majority of CHB and CHC patients produced virus-specific antibodies. The detectable anti-virus antibodies may stem from increased numbers of TFH cells in the CHB and CHC patients. More importantly, we found that the ratios of TFR to TFH in CHC, but not in CHB patients, were correlated negatively with the levels of serum anti-HCV antibodies in CHC patients (R = −0.4125, p = 0.0213). These data suggest that TFR cells may inhibit TFH cells and in turn down-regulate the ability of virus-specific B cells to produce antibodies. Therefore, there is a significant difference in regulating humoral responses between CHB and CHC patients. Interestingly, we found that the numbers of circulating IL-10^+^ Bregs and the ratios of TFR to TFH in HBeAg^+^ CHB patients were significantly higher than that in the HBeAg^−^ CHB patients. Given that HBeAg positivity in CHB patients has been considered a biomarker for HBV replication and active status of CHB, IL-10^+^ Bregs and TFR cells, together with Tregs, may be more powerful in down-regulating T cell immunity in the HBeAg^+^ CHB patients.

While Tregs functionally inhibit inflammation and protect the liver from many toxicants induced liver injury [47,48], we found that the numbers of circulating IL-10^+^ Bregs and TFR cells were correlated positively with the levels of serum ALT and AST in the CHB and CHC patients. The abnormally higher levels of ALT and AST have been considered liver damages it is unclear how significantly increased numbers of circulating IL-10^+^ Bregs and TFR cells are associated with the liver injury. IL-10 can inhibit inflammation and be a protector of liver injury by inducing Bcl-2 and Bclx_L_ expression [49,50], and there is no direct evidence that IL-10 causes liver injury. Notably, TGF-β1 can promote liver fibrosis through activation of the Smad signaling. It is possible that IL-10^+^ Bregs and TFR cells may secrete TGF-β1, which activates the Smad signaling to promote liver fibrosis and to induce other unknown factors, causing liver injury in those CHB and CHC patients. We are interested in further investigating how the increased numbers of circulating IL-10^+^ Bregs and TFR cells are associated with elevated levels of liver injury in CHB and CHC patients.

We recognized that our study had limitations, including small sample size, the single time-point measurement, and the lack of longitudinal follow-up, the function analysis of different subsets of T cells. Therefore, further longitudinal studies on the function of different subsets of T cells and B cells in a bigger population are warranted.

## Conclusion

Our data indicated that significantly increased numbers of circulating IL-10^+^ Bregs, TFR, TFH and Tregs in CHB and CHC patients and the numbers of IL-10^+^ Bregs and TFR cells were correlated positively with the levels of serum HBV DNA, HCV RNA, and ALT in CHB or CHC patients, respectively. To the best of our knowledge, this was the first report about the numbers of circulating IL-10^+^ Bregs and TFR in CHB and CHC patients. Our novel data suggest that increased numbers of IL-10^+^ Bregs and TFR, together with Tregs, may be associated with impaired eradication of HBV or HCV virus in CHB or CHC patients. Hence, our findings may provide new insights into regulation of virus-specific immune responses and virus replication during the pathogenic process of CHB and CHC.
